# Development and Characterization of a Dual Nanofibrillar Membrane of Polylactic Acid Loaded with Hydroxyapatite and Chlorhexidine for Guided Bone Regeneration

**DOI:** 10.3390/polym18182290

**Published:** 2026-09-19

**Authors:** Jose Roberto Sauma, Diego Batista-Menezes, Silvia Maldonado-Frías, Reynaldo Pereira Reyes, Mauricio Montero-Aguilar, Marco Antonio Alvarez-Perez, Daniel Chavarria-Bolanos

**Affiliations:** 1 Dentistry Postgraduate Program, Universidad de Costa Rica, San José 11501-2060, Costa Rica; josersauma@gmail.com (J.R.S.); mauricio.monteroaguilar@ucr.ac.cr (M.M.-A.); 2 National Laboratory of Nanotechnology, LANOTEC-CeNAT-CONARE, Pavas, San José 1174-1200, Costa Rica; dbatista@cenat.ac.cr (D.B.-M.); rpereira@cenat.ac.cr (R.P.R.); 3 Laboratorio de Bioingeniería de Tejidos, División de Estudios de Posgrado e Investigación, Facultad de Odontología, UNAM, Circuito Exterior s/n Cd. Universitaria, Mexico City C.P. 04510, Mexico; sylvymaf@yahoo.com.mx

**Keywords:** guided bone regeneration, polylactic acid membrane, chlorhexidine, electrospinning, hydroxyapatite

## Abstract

Guided bone regeneration requires membranes that combine structural stability, biocompatibility, and bioactive properties. This study aimed to develop a bilayer electrospun polylactic acid (PLA) membrane incorporating nanohydroxyapatite (nano-HA) and chlorhexidine (CHX). Membranes were fabricated by electrospinning at 15 kV and divided into four groups: PLA 10% (control), PLA 10%/CHX 0.2%, PLA 10%/nano-HA 10%, and a bilayer combining the CHX- and nano-HA-loaded formulations in separate monolayers, creating a bilayer nanofibrillar scaffold. We characterized microscopic structure, thermal behavior, and chemical composition using scanning electron microscopy, differential scanning calorimetry, thermogravimetric analysis, Fourier-transform infrared spectroscopy, and energy-dispersive X-ray spectroscopy. We evaluated cellular compatibility through cell adhesion and WST-1 metabolic activity assays. All groups exhibited randomly arranged nanofibrillar networks with comparable fiber morphology, while nano-HA-containing membranes showed particulate agglomerates. Thermal analyses indicated changes associated with material incorporation without evidence of major disruption of the PLA matrix, while chemical analyses confirmed incorporation of nano-HA and CHX. CHX-containing membranes did not compromise cell viability, whereas HA-containing membranes promoted favorable cell distribution and morphology. These findings demonstrate the feasibility of producing bilayer PLA membranes incorporating HA and CHX while maintaining suitable physicochemical characteristics and cellular compatibility.

## 1. Introduction

The rehabilitation of edentulous spaces with dental implants has substantially improved oral function and patients’ quality of life. However, predictable implant placement and long-term success depend on adequate bone volume to provide implant stability and support osseointegration [1]. When the available bone is insufficient, guided bone regeneration (GBR) is commonly employed to reconstruct deficient alveolar ridges. GBR uses a barrier membrane to isolate the defect from rapidly proliferating epithelial and connective tissue cells while maintaining a protected space that favors bone regeneration [2]. Similarly, guided tissue regeneration (GTR) applies the principle of selective cell exclusion to periodontal defects, facilitating repopulation by cells capable of regenerating periodontal and bone tissues [3].

Despite the clinical predictability of regenerative procedures, bacterial contamination and postoperative infection remain significant complications that can adversely affect regenerative outcomes [4]. Successful GBR therefore depends on appropriate wound management, including primary closure and the maintenance of a stable, protected regenerative environment [2,5]. Systemic antibiotics are frequently prescribed during implant placement and simultaneous GBR to reduce postoperative infection; however, evidence supporting their routine use remains controversial, with clinical studies reporting no significant improvement in several postoperative outcomes [4]. Local antimicrobial strategies may consequently provide an alternative or complementary approach. Chlorhexidine (CHX) is a broad-spectrum antiseptic commonly used in dentistry, and CHX rinses have been widely used after periodontal and implant surgery for postoperative plaque and biofilm control [6].

Autogenous bone remains an important grafting option for the reconstruction of alveolar bone defects because of its biological regenerative properties. Nevertheless, harvesting autogenous bone requires an additional surgical site and may result in donor-site complications, including postoperative pain, discomfort, sensory disturbances, and other morbidity [7]. These limitations have encouraged the development of bone tissue-engineering strategies in which biomaterial scaffolds provide a three-dimensional environment that supports tissue regeneration and may also serve as delivery systems for signaling molecules, such as growth factors [8].

Biomaterials play a central role in bone tissue engineering because their composition, architecture, mechanical properties, porosity, and degradation behavior can influence cell attachment, proliferation, differentiation, and subsequent tissue formation [9]. Ideally, scaffolds intended for regenerative applications should be biocompatible and biodegradable while maintaining sufficient structural integrity during tissue formation [10]. Poly(lactic acid) (PLA) is a biodegradable aliphatic polyester that has been extensively investigated for bone tissue engineering because of its biocompatibility, processability, and flexibility in scaffold design. Nevertheless, PLA has limitations, including low osteoconductivity and the potential for inflammatory reactions due to acidic degradation products, which encourages its combination with bioactive materials to improve performance in bone-regenerative applications [11].

Hydroxyapatite (HA), a calcium phosphate ceramic chemically similar to the mineral component of bone, is widely used in bone tissue engineering because of its biocompatibility and osteoconductive properties. Incorporating HA into PLA-based composites can modify their physicochemical and mechanical properties and enhance their suitability for bone-related applications [9,12]. Electrospinning enables the fabrication of highly porous nanofibrous structures with a high surface-area-to-volume ratio and morphological features that resemble certain aspects of the extracellular matrix. These characteristics favor cell–material interactions and make electrospun scaffolds attractive platforms for bone tissue engineering [13]. Electrospinning can also be used to incorporate therapeutic compounds into biodegradable polymeric membranes [14]. This technique has also been extensively investigated for incorporating antimicrobial compounds into polymeric fibers, and previous evidence indicates that selected bioactive agents can retain their antimicrobial activity after electrospinning [15]. Specifically, CHX has been successfully incorporated into electrospun cellulose acetate fibers, resulting in controlled release and antibacterial activity against representative oral pathogens [16]. Sustained CHX release and antibacterial activity have also been demonstrated in electrospun PLA fibers containing CHX particles [17]. These findings support the feasibility of incorporating CHX into electrospun polymeric matrices. To the best of our knowledge, only one previous study has combined nano-HA and emulsified CHX within the same electrospun nanofibrous scaffold [3]. In that formulation, however, both components were incorporated within the same fibrous matrix rather than spatially separated into distinct PLA layers. Collectively, these findings support the feasibility of incorporating CHX into electrospun polymeric matrices while highlighting the limited evidence regarding PLA-based architectures that spatially organize nano-HA and CHX into separate layers.

Recent research has increasingly focused on multifunctional nanofibrous scaffolds that combine components intended to support tissue regeneration with antimicrobial agents. For example, nanofibrous scaffolds incorporating nano-hydroxyapatite (nano-HA) and emulsified chlorhexidine (CHX) have demonstrated the feasibility of integrating an osteoconductive component and an antimicrobial agent within a single regenerative scaffold [3]. Similarly, the spatial separation of these components within bilayer or Janus membranes has already been established [18,19]. Therefore, the novelty of the present approach does not reside in the bilayer strategy or in the general concept of spatially separating regenerative and antimicrobial functions. Rather, it lies in the development of a specific PLA/nano-HA/CHX architecture in which nano-HA and CHX are incorporated into distinct electrospun PLA layers while maintaining a continuous nanofibrous structure and a stable interface between the two layers. This design enables the independent functionalization of each membrane surface: a nano-HA-containing surface intended to support cell–material interactions relevant to guided bone regeneration and a CHX-containing surface with potential localized antiseptic functionality. Because the biological response to CHX is concentration- and formulation-dependent, it is also necessary to determine whether its incorporation into this bilayer architecture affects the cytocompatibility of the resulting membrane. Accordingly, this study aimed to fabricate and characterize the PLA/nano-HA/CHX bilayer membrane and determine whether its spatially organized composition could be achieved without adversely affecting the morphology, physicochemical properties, or in vitro cytocompatibility of the polymeric matrix.

## 2. Materials and Methods

### 2.1. Hydroxyapatite Nanoparticles Characterization

#### 2.1.1. Dynamic Light Scattering (DLS)

The HA particles were synthesized and provided by the Materials Science and Engineering Research Center (CICIMA), University of Costa Rica. Five HA suspensions were independently prepared in distilled water at a final concentration of approximately 10% (*w*/*v*). Each suspension was vortexed for 5 min to promote particle redispersal and then equilibrated at 25 °C for 5 min before analysis. Hydrodynamic size distributions were determined using a nanoparticle analyzer based on dynamic light scattering (Nano Partica SZ-100V2, Horiba Scientific, Tokyo, Japan). Measurements were performed using a backscattering angle of 173° at recorded temperatures of 24.8–24.9 °C. Results were expressed as intensity-weighted size distributions. The Z-average hydrodynamic diameter, polydispersity index (PDI), and mean diameter and relative intensity-area contribution of each detected population were recorded.

#### 2.1.2. Nano-HA Scanning Electron Microscopy (SEM) Analysis

The surface and cross-sectional morphology of the hydroxyapatite nanoparticles were examined via SEM (JEOL JSM-6390LV, Tokyo, Japan) at an accelerating voltage of 15–20 kV. Prior to imaging, the samples were sputter-coated with a 20 nm gold layer for 180 s using a Denton Vacuum Desk V system to ensure adequate conductivity. Particle morphology was analyzed using ImageJ software (Version 1.54g, NIH, Bethesda, MD, USA).

#### 2.1.3. Energy-Dispersive X-Ray Spectroscopy (EDS)

Elemental composition was analyzed using an EDS detector (model 7582-M Inca X-sight Si; Oxford Instruments) coupled to the previously described SEM. Nano-HA were mounted on aluminum SEM stubs using conductive carbon tape, without gold coverage. The SEM was operated at an accelerating voltage of 15 kV, with a working distance of 10 mm. EDS spectra were collected from multiple regions of interest across each sample to ensure representative elemental analysis.

### 2.2. Membrane Synthesis by Electrospinning

PLA pellets (2003D, Promaplast, Mexico City, Mexico) were dissolved at a concentration of 10% (*w*/*v*) in a chloroform/ethanol solvent system (3:1, *v*/*v*) under continuous stirring at 300 rpm for 24 h to prepare the control PLA solution. For the experimental formulations, nano-HA and CHX were separately added to their respective PLA solutions before stirring. Each formulation was then stirred continuously at 300 rpm for 24 h to ensure homogeneous dispersion in the polymer solution.

Electrospinning was performed horizontally using a syringe-pump system equipped with a 21-gauge needle (internal diameter: 0.508 mm). A potential difference of 15 kV was applied between the needle tip and a grounded metallic collector positioned at a tip-to-collector distance of 10 cm. The polymer solutions were delivered at a constant flow rate of 1 mL/h. Environmental conditions were maintained at a room temperature of 24 ± 2 °C, and controlled room humidity of 65 ± 2%.

**Figure 1:**
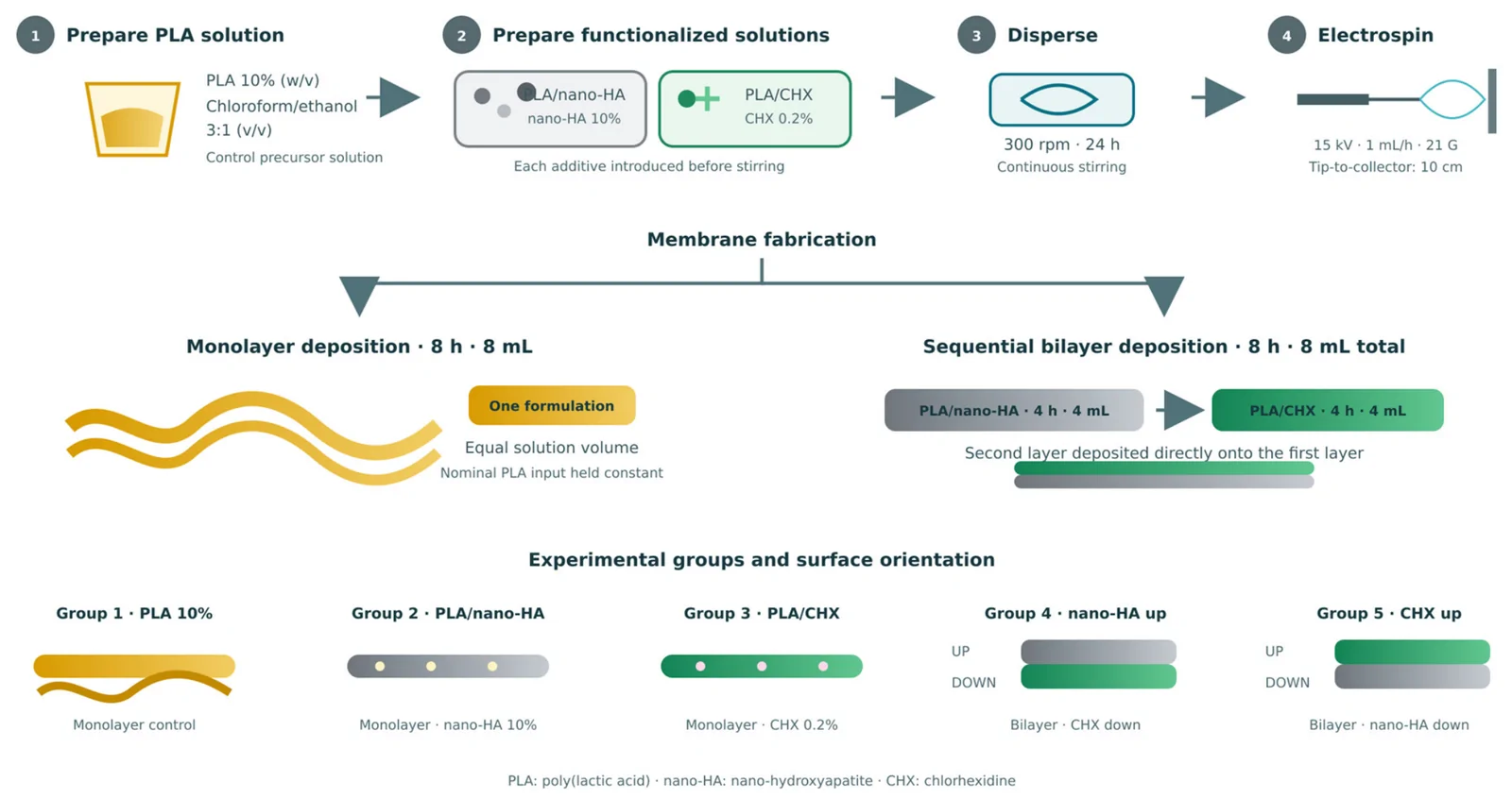
Schematic representation of the fabrication of PLA-based monolayer and bilayer electrospun membranes. PLA, PLA/nano-HA, and PLA/CHX precursor solutions were prepared and electrospun under standardized processing conditions. Monolayer membranes were produced by electrospinning the corresponding formulation for 8 h. The bilayer membrane was fabricated by sequentially depositing the PLA/nano-HA layer for 4 h, followed by the PLA/CHX layer for an additional 4 h. The resulting membrane was evaluated with either the nano-HA-containing or CHX-containing surface facing upward. PLA: poly(lactic acid); nano-HA: nano-hydroxyapatite; CHX: chlorhexidine.

For the monolayer membranes, we electrospun 8 mL of the corresponding polymer solution (PLA 10%, PLA 10% (*w*/*w*) nano-HA, and PLA 0.2% (*w*/*w*) CHX) for 8 h. To fabricate the bilayer membrane, the PLA/nano-HA solution was first electrospun for 4 h, corresponding to a deposited solution volume of 4 mL. The 0.2% CHX solution was subsequently electrospun for an additional 4 h directly onto the previously deposited PLA/nano-HA layer. This sequential deposition produced a bilayer membrane comprising a PLA/nano-HA-oriented surface and an opposing PLA/CHX-oriented surface. We maintained the total processing time and solution volume at 8 h and 8 mL, respectively, for both monolayer and bilayer membranes. Consequently, the nominal PLA input was equivalent among the groups, although the actual deposited mass was not determined gravimetrically. Figure 1 shows a schematic of the fabrication procedure, membrane orientation, and experimental groups.

### 2.3. Membranes Physicochemical Characterization

#### 2.3.1. Membranes SEM Analysis

The surface and cross-sectional morphologies of the electrospun scaffolds were examined using SEM, as previously described. Micrographs were obtained to analyze the outer and inner surfaces of the membranes and the cross-sectional width. Fiber diameter and total membrane thickness were quantified using ImageJ software (Version 1.54g, NIH, Bethesda, MD, USA). Five independently fabricated membranes were evaluated per experimental group. Ten fiber-diameter measurements were obtained from each membrane, yielding 50 measurements per group. Membrane thickness was similarly determined from ten randomly selected locations in cross-sectional micrographs of each membrane. To obtain the images for analysis, individual membranes were imaged at 2000×.

#### 2.3.2. Differential Scanning Calorimetry (DSC)

Calorimetric behavior was characterized using differential scanning calorimetry (DSC) (DSC-4000, Perkin-Elmer Inc., Waltham, MA, USA). Samples (5.0 ± 0.5 mg) of pure PLA, nano-HA, and CHX powders, along with representative specimens from each experimental group (*n* = 3 per group; total *n* = 12), were encapsulated in sealed aluminum crucibles. Thermal scans were performed from 25 °C to 200 °C at a constant heating rate of 10 °C/min. Data were processed using Pyris software (version 13.4.0.0036, PerkinElmer Inc., Waltham, MA, USA) to determine the glass transition temperature (T_g-rel_), melting temperature (T_m_), and cold crystallization point (T_cc_), and the corresponding transition enthalpies (ΔH_g-rel_, ΔH_cc_, and ΔH_m_) by peak integration. All thermograms were obtained from the first heating cycle.

#### 2.3.3. Thermogravimetric Analysis (TGA)

The thermal stability and decomposition profiles of the samples were evaluated using thermogravimetric analysis (TGA-4000, PerkinElmer Inc., Waltham, MA, USA). Specimens (5.0 ± 0.5 mg) of pure PLA, nano-HA, and CHX, along with three samples from each experimental group (*n* = 12 total), were placed in the analyzer. The samples were initially held at an isothermal base temperature of 25 °C for 3 min to ensure stabilization. Subsequently, a constant heating ramp was applied from 25 °C to 600 °C at 20 °C/min under a controlled atmosphere. The onset temperature of degradation (T_o_) and the inflection point (T_p_) were determined. Data acquisition and processing were performed using Pyris software (version 13.4.0.0036, PerkinElmer Inc., Waltham, MA, USA).

#### 2.3.4. Fourier Transform Infrared Spectroscopy (FTIR)

Chemical characterization of pure PLA, nano-HA, and CHX powders, as well as representative specimens from each experimental group (*n* = 4 total), was conducted using an FT-IR Nicolet iS50 spectrometer (Thermo Fisher Scientific, Waltham, MA, USA) to obtain infrared absorption spectra. We acquired and evaluated the data using OMNIC 9 software (Omnic series 2.14, Thermo Fisher Scientific, Waltham, MA, USA). The spectra were baseline-corrected and linearized to identify characteristic functional group signals. Comparative analyses confirmed the incorporation of nano-HA and CHX into the scaffolds and evaluated potential chemical interactions or structural changes in the polymeric matrix.

#### 2.3.5. Energy-Dispersive Spectroscopy (EDS)

We mounted one sample from each evaluated scaffold condition (*n* = 5 specimens total) on aluminum SEM stubs using conductive carbon tape. EDS analysis was performed as previously described, ensuring sufficient X-ray counts while avoiding sample damage. EDS spectra were collected from multiple regions of interest across each sample to ensure representative elemental analysis. A comparative analysis was performed on the obtained spectra.

### 2.4. Cellular Biocompatibility

#### 2.4.1. Cell Adhesion

Human fetal osteoblasts (hFOB 1.19, ATCC CRL-11372; ATCC, Manassas, VA, USA) were utilized to evaluate cell adhesion and viability. The cells were cultured in 75 cm^2^ flasks using a 1:1 mixture of Ham’s F12 and Dulbecco’s Modified Eagle Medium (DMEM), supplemented with 10% fetal bovine serum (FBS), 2.5 mM L-glutamine, and 1% penicillin/streptomycin. Cultures were maintained at 34 °C (the permissive temperature for this cell line) in a humidified atmosphere with 5% CO_2_. Cells from passages 2–6 were used for all experimental procedures. We sterilized scaffold discs by immersing them in 70% ethanol for 30 min, followed by three rinses with sterile phosphate-buffered saline (PBS) and a final rinse with deionized water, then air-dried them under sterile conditions. For the cell adhesion assay, hFOB cells were seeded onto the discs at a density of 1 × 10^4^ cells/mL and incubated for 4 and 24 h. After incubation, remove non-adherent cells by gentle PBS washing. We fixed adherent cells with 4% paraformaldehyde and stained them with 0.1% crystal violet. The incorporated dye was subsequently solubilized with 0.1% sodium dodecyl sulfate (SDS), and the absorbance was measured at 545 nm (using a microplate reader) as an indirect quantification of cell attachment.

#### 2.4.2. Metabolic Activity Assay

The metabolic activity of cells cultured on the experimental scaffolds was evaluated using the WST-1 colorimetric assay (Roche Diagnostics, Mannheim, Germany). hFOB 1.19 cells were seeded onto the material discs at a density of 1 × 10^4^ cells/mL and cultured for 2, 4, and 6 days. At each designated time point, the samples were incubated with the WST-1 reagent for 4 h. Absorbance was subsequently measured at 450 nm using a microplate reader. The culture medium was refreshed every 72 h throughout the experimental period. The absorbance values were used as a relative indicator of cellular metabolic activity and scaffold cytocompatibility.

#### 2.4.3. Qualitative Fluorescence Microscopy Analysis

For fluorescence analysis, PKH67 Green fluorescent cell linker (PKH67, Sigma-Aldrich, St. Louis, MO, USA) was used. Briefly, hFOB cells were washed before seeding onto the membrane scaffold, resuspended in diluent C, and incubated with the dye solution for 5 min according to the manufacturer’s instructions. Once hFOB cells were stained, the cells were seeded at a 1 × 10^4^ cell/sample, and after 24 h, the membrane scaffolds were observed with a fluorescence LED microscope (Amscope, Irvine, CA, USA). We qualitatively evaluated fluorescence micrographs to assess osteoblast distribution and morphology across the different membrane formulations. We reviewed four microscopic fields for each experimental condition. The qualitative assessment considered the presence and distribution of fluorescent cells, apparent cellular coverage, and morphological features associated with cell attachment and spreading, including polygonal or elongated cell bodies and cytoplasmic extensions. We selected one representative micrograph from each group.

### 2.5. Statistical Analysis

For the DSC and TGA data, we evaluated differences among groups using one-way analysis of variance (ANOVA), followed by Tukey’s post hoc test when statistically significant differences were detected. We assessed fiber diameter and transverse membrane thickness using ten measurements from each of five independently fabricated and mounted membranes per experimental group. We averaged the ten measurements for each membrane, with each membrane as the experimental unit. We evaluated normality using the Shapiro–Wilk test. Differences in fiber diameter among the five experimental groups were analyzed using the Kruskal–Wallis test, followed by Dunn’s post hoc test with adjustment for multiple comparisons. We evaluated differences in membrane thickness among the four experimental groups using the Kruskal–Wallis test, followed by pairwise Mann–Whitney U tests with Bonferroni correction because one group did not meet the normality assumption. We set statistical significance at *p* < 0.05. Descriptive results are presented as mean ± standard deviation. The assessed data normality and variance homogeneity using the Shapiro–Wilk test, and to determine significant differences among groups, we performed one-way ANOVA followed by Tukey’s post hoc analysis. All quantitative data were expressed as the arithmetic mean ± standard error of the mean, with a significance level of *p* ≤ 0.05.

## 3. Results

### 3.1. Nano-HA Characterization

**Figure 2:**
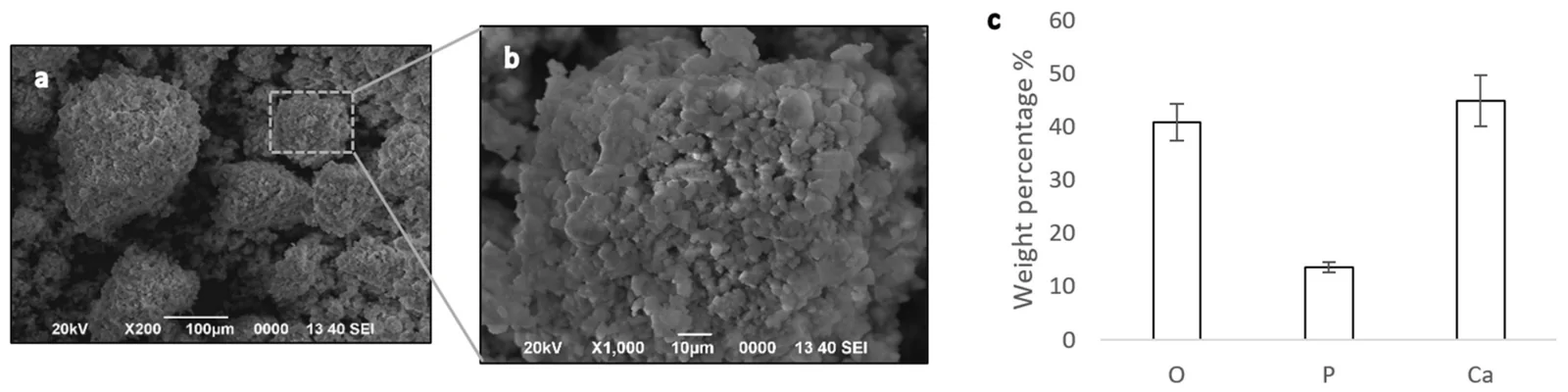
Hydroxyapatite nanoparticles characterization. Scanning electron microscopy (SEM) at 200× (**a**) and 1000× (**b**), and (**c**) elemental analysis (EDS).

SEM micrographs at increasing magnifications revealed predominantly rounded HA agglomerates with rough, highly textured surfaces (Figure 2a,b). At 5000× magnification, these micrometer-scale agglomerates showed hierarchical organization with progressively finer particulate substructures. Calibrated ImageJ measurements confirmed the presence of identifiable structures with dimensions below 100 nm within the larger agglomerates. DLS revealed highly polydisperse and predominantly multimodal HA dispersions. Across the five measurements, the Z-average hydrodynamic diameter ranged from 324.3 to 502.0 nm, with an overall mean of 401.8 ± 75.1 nm. The PDI ranged from 0.730 to 0.825 (0.778 ± 0.040), indicating broad and heterogeneous size distributions. Intensity-weighted analysis detected populations below 200 nm in all measurements, including some hydrodynamic populations below 100 nm. Larger populations are consistent with the agglomerated structures observed by SEM.

### 3.2. Electrospun Scaffolds Characterization

**Figure 3:**
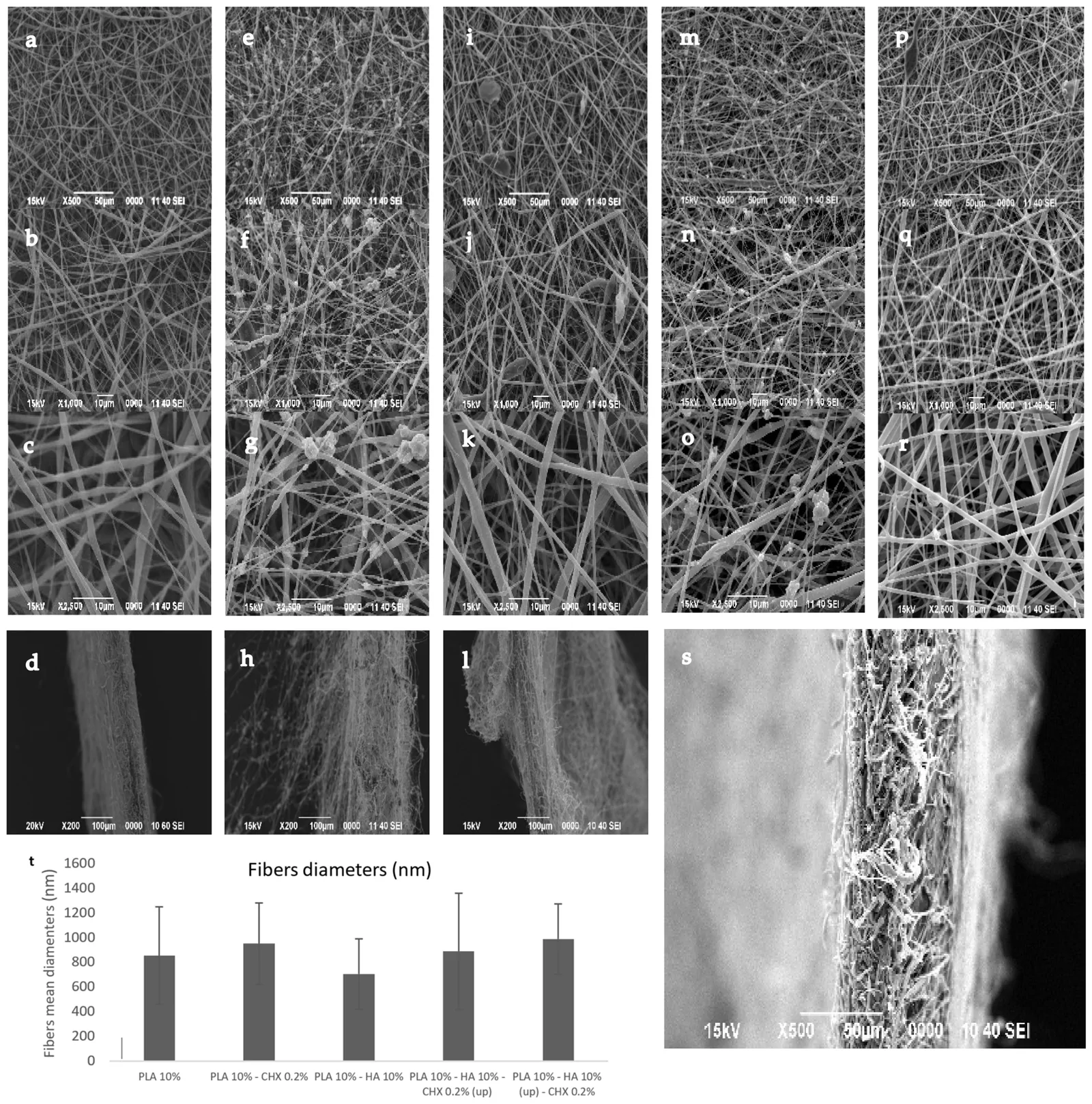
SEM of the obtained membranes. Group A PLA 10% (**a**–**d**); Group B PLA 10%/nano-HA 10% (**e**–**h**); Group C PLA 10%/CHX 0.2% (**i**–**l**); Group D.1 PLA 10%/nano-HA 10%/CHX 0.2% (nano-HA on top) (**m**–**s**); and Group D.2 PLA 10%/nano-HA 10%/CHX 0.2% (nano-HA on bottom) (**p**–**s**). (**t**) Fiber diameter mean values (nm).

SEM micrographs of all scaffold groups are presented in Figure 3. The images revealed the formation of interconnected fibrous networks with randomly oriented fibers and porous architectures in all samples. Group A (Figure 3a–d) showed smooth, continuous fibers with a homogeneous morphology throughout the scaffold. In Group B (Figure 3e–h), particulate structures and small agglomerates were observed distributed along the fibers and at fiber intersection points. Group C (Figure 3i–l) maintained a predominantly fibrous morphology, with smooth fibers and only a limited number of isolated structures within the matrix. Group D (Figure 3m–s) exhibited a dual-surface morphology depending on the side analyzed. The nano-HA surface (left side of s) showed characteristics similar to Group B, whereas the CHX surface (right side of s) resembled Group C. These observations suggest the presence of distinct surface characteristics within the scaffold structure. Figure 3t shows the mean fiber diameter of the electrospun membranes.

Fiber diameters ranged from approximately 670 to 990 nm, with the PLA 10–nano-HA 10% group showing the lowest value and the PLA 10–CHX 0.2–nano-HA 10% (HA up) group the highest. Statistical analysis revealed significant differences among groups (Kruskal–Wallis, *p* = 0.0043), with a significant increase in fiber diameter observed between the PLA 10%/nano-HA 10% and PLA 10%/CHX 0.2%/nano-HA 10% (HA up) groups (Dunn’s post hoc test, *p* = 0.0015). These results indicate that the incorporation and distribution of nano-HA and CHX influenced fiber formation during electrospinning.

Cross-sectional membrane thickness differed significantly among the experimental groups. Because the PLA 10%/nano-HA 10% group did not satisfy the normality assumption according to the Shapiro–Wilk test (*p* < 0.05), comparisons were performed using non-parametric tests. The Kruskal–Wallis test revealed a significant overall difference among the formulations (H = 35.53, *p* < 0.000001). The PLA 10%/nano-HA 10% membrane exhibited the greatest mean thickness (148.30 ± 12.77 µm), followed by PLA 10% (95.00 ± 5.10 µm), PLA 10%/CHX 0.2% (82.80 ± 6.78 µm), and PLA 10%/CHX 0.2%/nano-HA 10% (69.30 ± 2.63 µm). Pairwise Mann–Whitney U tests with Bonferroni correction showed significant differences between all group pairs, as indicated by the different uppercase letters in Table 1.

### 3.3. Physicochemical Characterization

**Figure 4:**
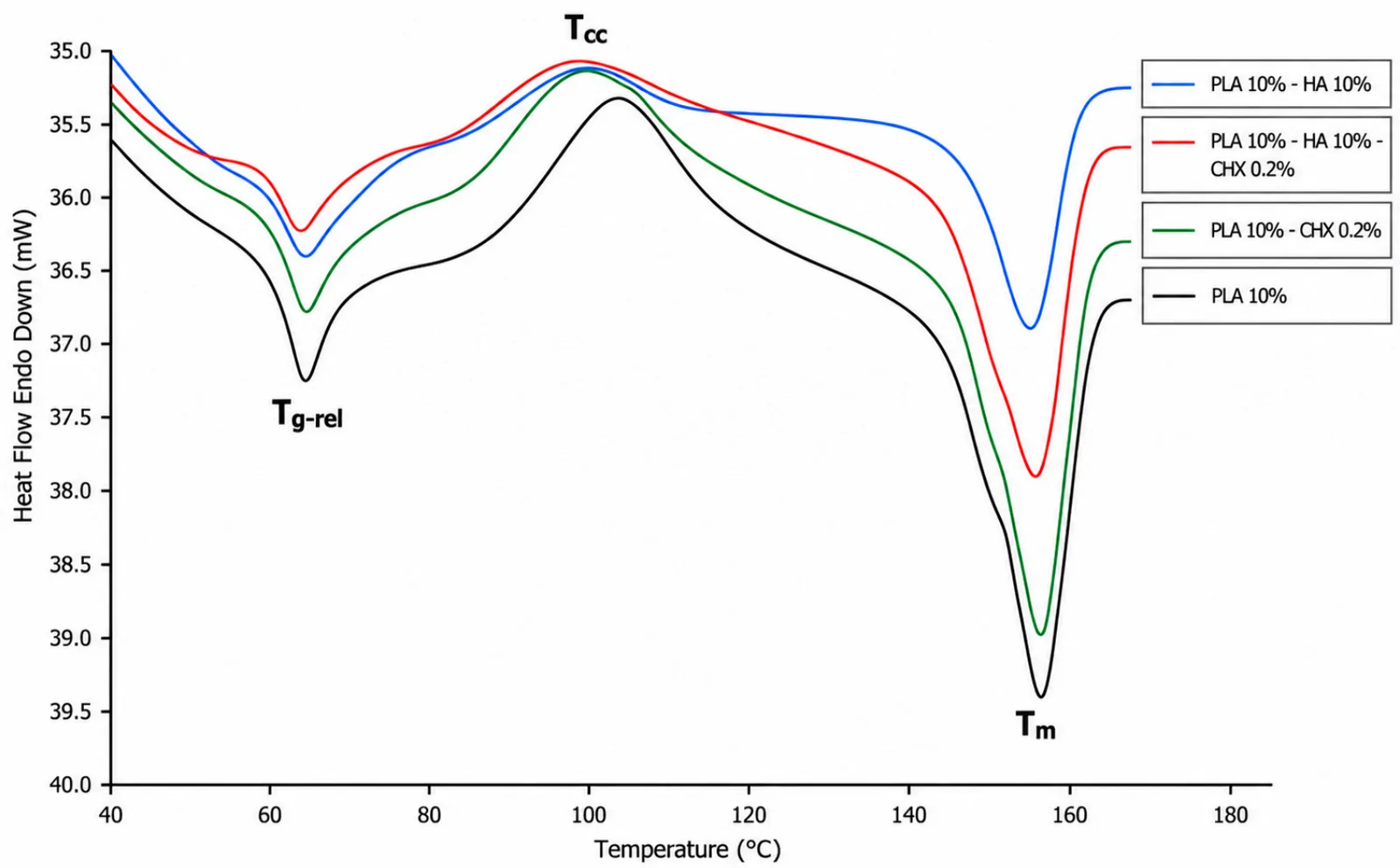
DSC thermograms compilation. T_g-rel_: enthalpic-relaxation event associated with the PLA glass-transition region. T_cc_: temperature of cold-crystallization. T_m_: melting point.

The DSC thermograms of all scaffold formulations showed similar thermal profiles (Figure 4), characterized by three main thermal events: a transition accompanied by an enthalpic-relaxation peak within the PLA glass-transition region (T_g-rel_) at approximately 60–70 °C. An exothermic cold-crystallization event (T_cc_) occurred between approximately 95 and 110 °C, followed by an endothermic melting signal (T_m_) between approximately 150 and 160 °C. No statistically significant differences were observed among the groups in the T_g-rel_ or T_m_. Differences were detected in T_cc_, indicating that the incorporation of CHX and/or nano-HA influenced the thermal behavior associated with cold crystallization. (Table 2).

Analysis of the integrated DSC peaks revealed no significant differences among formulations in the enthalpy associated with the T_g-rel_ (ΔH_g-rel_). Conversely, the crystallization enthalpy (ΔH_cc_) was significantly lower for PLA/nano-HA (25.38 ± 10.07) and PLA/CHX/nano-HA (47.49 ± 15.90) compared with PLA alone (88.33 ± 1.58). At the same time, PLA/CHX (58.62 ± 18.08) showed intermediate values without significant differences from the other groups. For the melting transition, the melting enthalpy (ΔH_m_) was significantly lower for PLA/nano-HA (79.23 ± 2.45) than for PLA (148.33 ± 7.13), PLA/CHX (165.42 ± 33.52), and PLA/CHX/nano-HA (160.91 ± 12.59) (Table 3).

Thermogravimetric analysis showed that the onset temperatures ranged from approximately 327 to 339 °C across the different formulations. PLA 10% exhibited the highest mean To (338.70 ± 7.75 °C), followed by PLA/nano-HA (327.76 ± 12.07 °C), PLA/CHX (327.65 ± 10.92 °C), and PLA/CHX/nano-HA (326.89 ± 26.37 °C). However, no statistically significant differences were detected among groups (*p* = 0.774). Table 4 shows the inflection points. Although lower mean Tp values were observed for the nano-HA-containing formulations, no statistically significant differences were detected among groups (*p* = 0.556).

**Figure 5:**
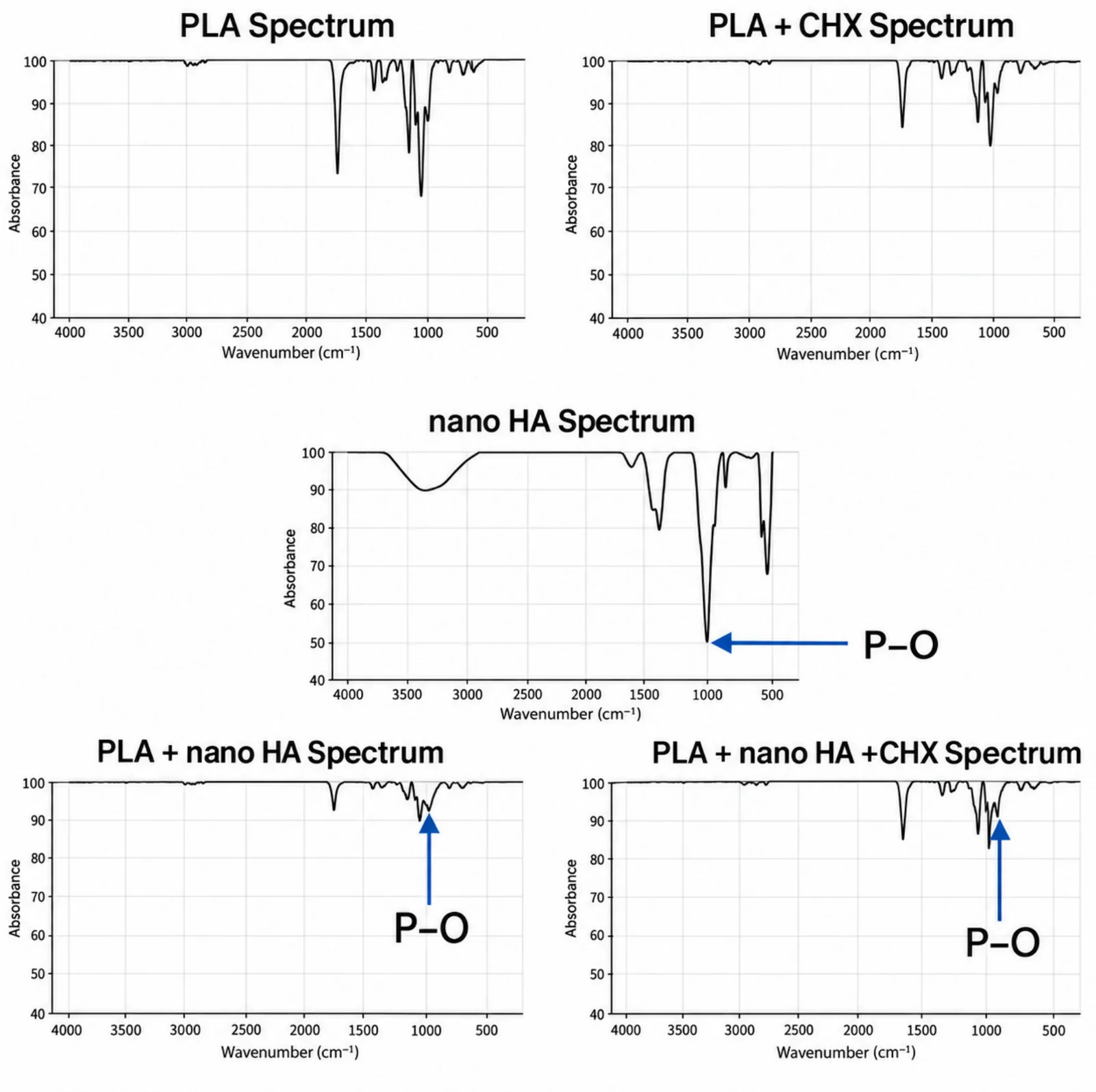
Fourier transform infrared spectroscopy (FTIR) for PLA, HA, and CHX.

The FTIR spectra of all formulations are presented in Figure 5. All groups showed characteristic PLA absorption bands. The PLA/CHX scaffold exhibited a spectral profile similar to that of PLA alone, with no clearly distinguishable additional bands attributable to CHX. In contrast, the HA-containing formulations exhibited additional phosphate-related (P–O) absorption bands in the 1000–1100 cm^−1^ region, consistent with the presence of hydroxyapatite. These signals were observed in both the PLA/nano-HA and PLA/nano-HA/CHX scaffolds.

**Figure 6:**
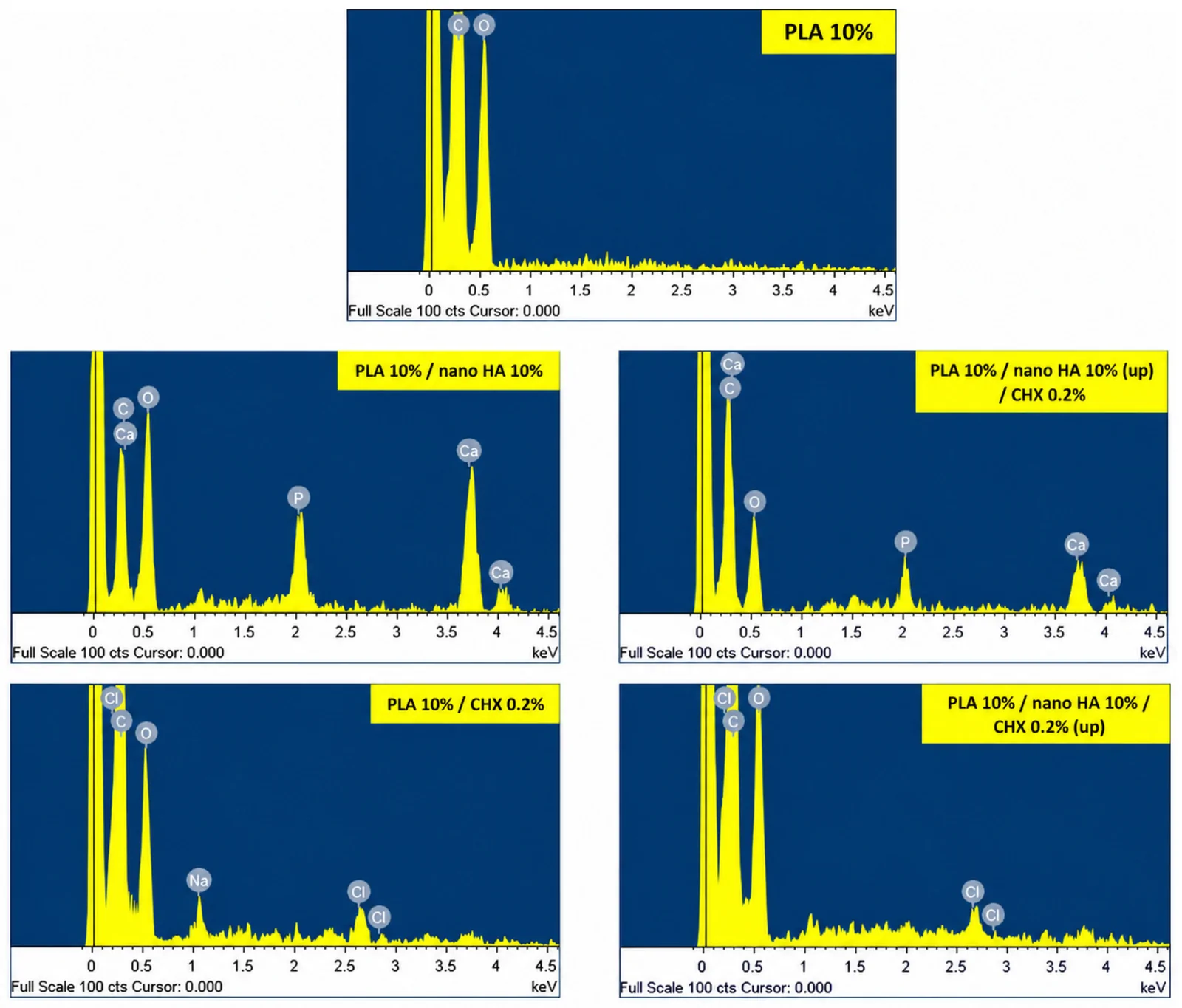
Energy-Dispersive Spectroscopy (EDS) for all groups.

Elemental analysis by EDS further supported the expected composition of the different scaffold formulations (Figure 6). The PLA scaffold exhibited only carbon (C) and oxygen (O) signals, whereas chlorine (Cl) was detected in the PLA/CHX scaffold, consistent with the incorporation of CHX. The PLA/HA scaffold exhibited phosphorus (P) and calcium (Ca) signals, supporting the presence of hydroxyapatite. In the PLA/HA/CHX scaffold, EDS analysis detected Cl in the PLA/CHX region and P and Ca in the PLA/HA region, confirming the presence of the corresponding components within the respective regions of the combined scaffold.

### 3.4. Biocompatibility Results

**Figure 7:**
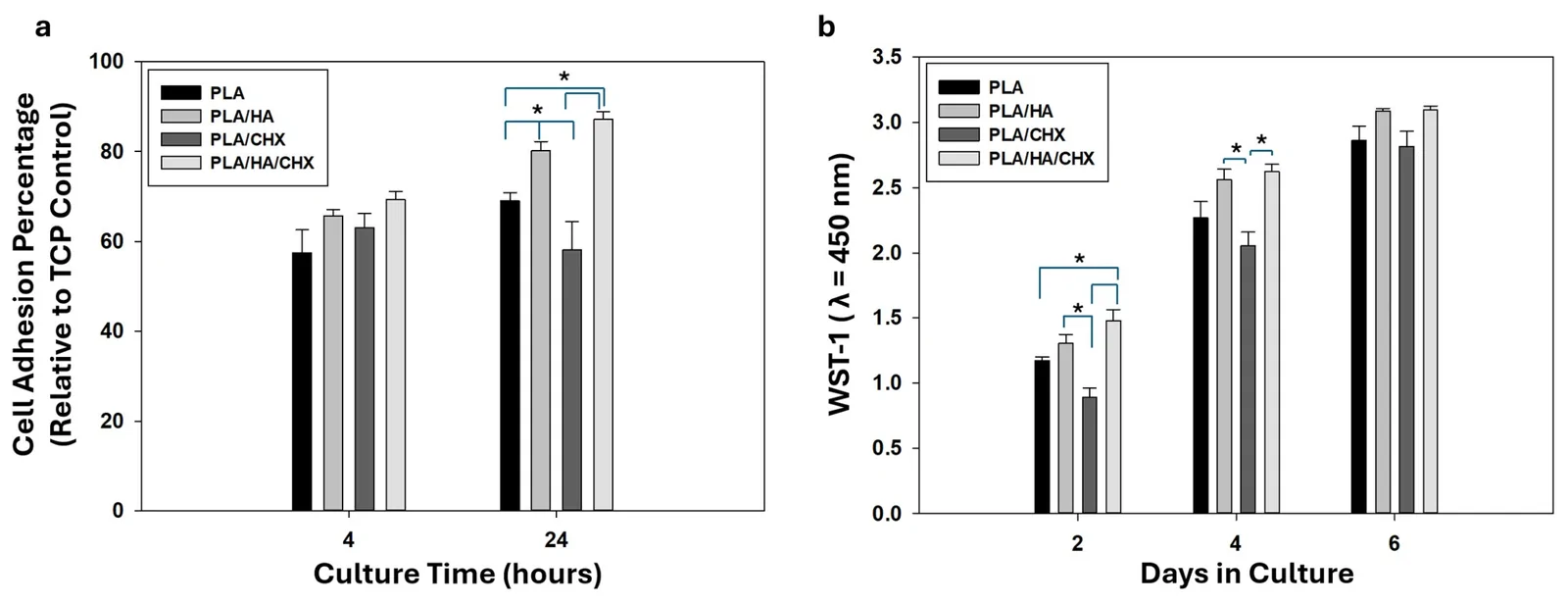
Cellular biocompatibility assays. (**a**) Cell adhesion; (**b**) Cell metabolic activity assay. An asterisk (*) means that scaffolds showed statistical significance (*p* < 0.05).

Cell adhesion results at 4 h and 24 h are presented in Figure 7a. After 4 h of culture, adhesion percentages ranged from approximately 58% to 70%, with the PLA/HA/CHX group exhibiting the highest value, followed by PLA/CHX and PLA/HA, whereas PLA exhibited the lowest cell adhesion. At 24 h, cell adhesion increased in the PLA, PLA/HA, and PLA/HA/CHX groups, reaching approximately 69% to 87%. In contrast, the PLA/CHX group showed a slight decrease in adhesion at 24 h compared with the 4 h. Among all formulations, PLA/HA/CHX and PLA/HA showed the highest cell adhesion at both time points (Figure 7a).

Cell metabolic activity, assessed by the WST-1 assay, increased over time in all experimental groups (Figure 7b). At day 2, WST-1 values ranged from approximately 0.9 to 1.5, with the PLA/HA/CHX group exhibiting the highest value. By day 4, all groups showed increased metabolic activity, reaching values between approximately 2.0 and 2.6. After 6 days of culture, the highest WST-1 values were observed for the PLA/HA and PLA/HA/CHX groups (≈3.1), followed by PLA and PLA/CHX (≈2.8–2.9). Overall, all formulations demonstrated a progressive increase in WST-1 absorbance throughout the culture period.

**Figure 8:**
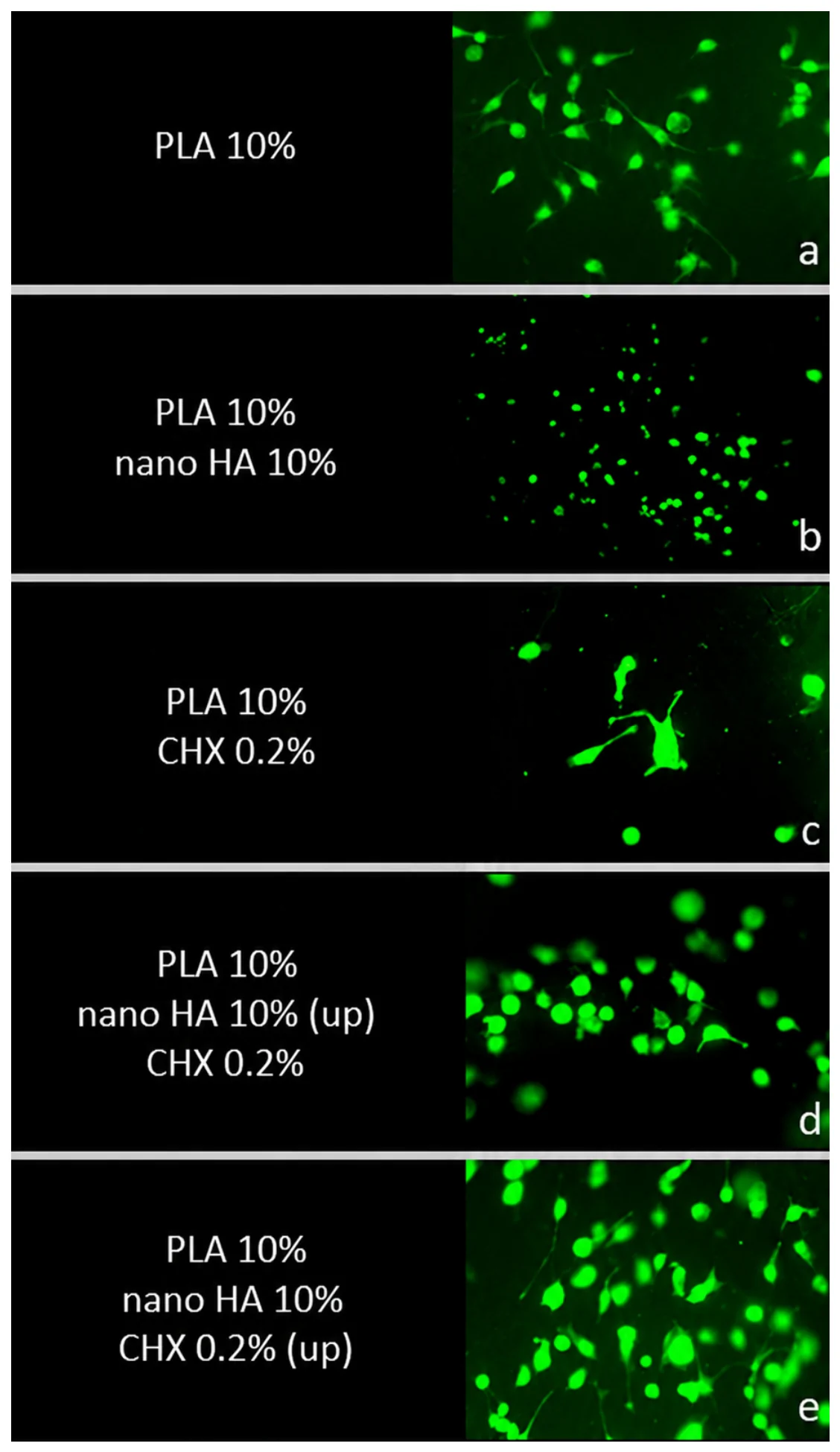
Representative fluorescence micrographs showing the qualitative osteoblast response to the evaluated electrospun membranes. (**a**) PLA 10%; (**b**) PLA 10%/nano-HA 10%; (**c**) PLA 10%/CHX 0.2%; (**d**) bilayer PLA 10%/nano-HA 10%/CHX 0.2% membrane with the nano-HA-containing surface facing upward; and (**e**) bilayer membrane with the CHX-containing surface facing upward. Fluorescent cells were observed on all membrane formulations. Nano-HA-containing groups frequently showed areas of greater apparent cellular coverage and cells displaying spread or elongated morphologies.

Fluorescence microscopy showed the presence of attached osteoblasts on all evaluated membrane formulations. Cells exhibited variable morphologies, ranging from rounded profiles to elongated or polygonal cell bodies with visible cytoplasmic extensions, indicating that the different membrane surfaces permitted cellular attachment and spreading. The PLA and PLA/CHX membranes supported the presence of fluorescent cells, although their distribution and apparent surface coverage varied among the examined fields (Figure 8).

Qualitatively, the nano-HA-containing formulations frequently exhibited areas with greater apparent cellular coverage and numerous cells displaying spread or elongated morphologies. This pattern was observed both in the single-layer PLA/nano-HA membrane and in the bilayer configurations. Overall, the fluorescence findings are consistent with a favorable osteoblast response and support the cytocompatibility results obtained using WST-1.

## 4. Discussion

The present study demonstrated that it was possible to create a dual electrospun PLA-based scaffold incorporating nano-HA and CHX while maintaining their individual distributions within the same matrix. Overall, incorporation of these components preserved the characteristic interconnected fibrous architecture of the electrospun PLA while inducing formulation-dependent changes in fiber morphology, diameter, and physicochemical behavior. Chemical and physical analyses confirmed the presence of nano-HA and CHX, while thermal analyses showed that their incorporation influenced PLA crystallization and transition enthalpies without significantly affecting the temperatures associated with its main melting transitions or thermal degradation. Notably, the resulting scaffolds exhibited favorable cellular adhesion and metabolic activity.

PLA was selected as the polymeric matrix due to its excellent biocompatibility, biodegradability, favorable mechanical properties, and ease of electrospinning into nanofibrous scaffolds. These characteristics have made PLA one of the most extensively investigated synthetic polymers for bone tissue-engineering applications [11,20,21]. Previous studies have employed 10 wt% nano-HA in electrospun PLA-based fibers and reported changes in their thermal and mechanical properties while maintaining favorable osteoblast attachment, supporting the selection of this concentration for the initial fabrication of bone tissue-engineering scaffolds [20]. In the present study, the nominal 10 wt% nano-HA loading preserved the overall fibrous architecture, although we observed localized particle-rich regions and aggregates. The biological and physicochemical implications of these localized aggregates were not investigated and therefore cannot be considered inherently unfavorable, as they may influence surface interactions, cellular responses, or release behavior. Nevertheless, if a more homogeneous particle distribution and reduced aggregation are desired, future studies should evaluate lower nano-HA concentrations and/or alternative dispersion strategies. On the other hand, CHX was incorporated at 0.2% because it is widely used in dentistry, provides broad-spectrum antimicrobial activity, and maintains acceptable biocompatibility when delivered locally via biomaterial-based systems [6,14].

Regarding the nano-HA, the combined SEM and DLS findings indicate that the HA material exhibited a hierarchical and highly agglomerated organization. Calibrated measurements of the SEM micrograph confirmed the presence of identifiable particulate substructures below 100 nm within larger micrometer-scale agglomerates, supporting the use of the term nano-HA to describe the finer constituent structures. In contrast, DLS yielded high PDI values and multimodal intensity distributions because it measured the hydrodynamic dimensions of the complete dispersed entities, including agglomerates. Furthermore, intensity-weighted DLS is disproportionately influenced by larger scattering entities. Thus, the Z-average values should not be interpreted as the physical diameter of the individual nano-HA structures. Rather, the results demonstrate the coexistence of sub-100 nm particulate features and larger agglomerated structures.

The SEM images of the PLA 10% scaffold (Figure 3a–d) showed uniform, clean nanofibers with smooth surfaces and random orientation. The scaffold exhibited a homogeneous structure and well-defined fiber continuity, as reported in previous investigations of electrospun PLA scaffolds [22]. The PLA 10%/nano-HA 10% scaffold (Figure 3e–h) exhibited nanofibers with a cotton-like structure and local aggregates. These features were also observed in the cross-section, suggesting the successful incorporation of nano-HA into the PLA scaffold [12,20]. The incorporation of 0.2% CHX did not produce noticeable alterations in the nanofibrous morphology of the electrospun PLA scaffold. The fibers remained smooth, continuous, and bead-free, closely resembling those of the control PLA group, indicating that the selected CHX concentration did not adversely affect fiber formation. Similar observations have been reported for electrospun antimicrobial scaffolds, where incorporation of antimicrobial agents can preserve the structural integrity of the nanofibrous network while allowing drug incorporation within the polymer matrix [14,23].

The dual-layer PLA/nano-HA/CHX scaffold maintained a well-defined nanofibrous architecture while exhibiting distinct surface morphologies depending on the side examined. When the nano-HA-containing surface was exposed (Figure 3m–o), the fibers displayed nanoparticle aggregates and increased surface roughness, closely resembling the PLA/nano-HA scaffold. In contrast, the CHX-loaded surface (Figure 3p–s) exhibited smooth, homogeneous fibers comparable to the PLA/CHX group, with no visible particulate deposits. These findings indicate that the dual electrospinning process successfully preserved the individual morphological characteristics of each layer without compromising fiber continuity or overall scaffold integrity. Unlike conventional homogeneous composite scaffolds, the present dual-layer design spatially separates the osteoconductive and antimicrobial components, potentially allowing each surface to interact differently with the surrounding tissues while minimizing interference between their respective functions [24]. The concept of bifunctional membranes that combine distinct biological functions has been reported previously [18]. Notably, the interface between the two layers showed apparent morphological continuity at the layer interface by the polymeric nanofibers, indicating effective structural integration of both components of the scaffold. This interfacial continuity may help maintain scaffold integrity and minimize layer separation under biological conditions.

The DSC analysis demonstrated that the incorporation of nano-HA, CHX, and their combination did not significantly affect T_g-rel_ or T_m_. However, the transition enthalpy associated with T_m_ was significantly reduced in the PLA/nano-HA scaffold, indicating that the polymer’s primary melting behavior was preserved. Similar findings have been reported for PLA composites containing HA, where incorporation of the inorganic phase can modify thermal behavior without substantially altering the polymer’s characteristic melting transition [15]. In contrast, significant reductions in T_cc_ were observed in the nano-HA- and CHX-containing groups when compared to PLA 10%, indicating that both additives influenced the crystallization behavior of PLA. This behavior may be attributed to interactions between the polymer chains and the incorporated particles or molecules, which can influence chain mobility, nucleation, and crystal formation [20,21]. Likewise, the lower cold-crystallization and melting enthalpies observed for the PLA/nano-HA scaffold indicate that nano-HA affected the corresponding thermal events. Because the degree of crystallinity was not calculated, these enthalpy changes should not be interpreted as direct evidence of differences in PLA crystallinity.

The thermogravimetric behavior of the scaffolds showed that the incorporation of nano-HA modified the thermal degradation profile of the composite. This behavior is consistent with previous studies of PLA/HA composites, in which the thermally stable inorganic HA phase influences the thermal response of the material during degradation of the PLA matrix [20,21]. However, because the membrane analyses were conducted only up to 600 °C and did not reach a stable terminal plateau, the remaining mass could not be attributed exclusively to HA or used to estimate the actual inorganic fraction. In contrast, the incorporation of CHX at a low concentration had a limited effect on the degradation profile, suggesting that CHX could be incorporated without markedly altering the thermal behavior of the polymeric matrix. The bilayer PLA/nano-HA/CHX scaffold retained the principal thermal transitions and degradation temperatures of PLA despite the incorporation of both components, indicating that functionalization did not substantially compromise the overall thermal stability of the polymeric matrix. In this context, DSC and TGA provide complementary information for the physicochemical characterization of functionalized scaffolds. Changes in thermal transitions, transition enthalpies, and degradation profiles may provide indirect evidence of component incorporation while allowing an assessment of whether functionalization alters the thermal characteristics of the polymeric matrix.

FTIR analysis confirmed that the incorporation of nano-HA and CHX did not substantially alter the characteristic chemical structure of PLA, as evidenced by preservation of its main absorption bands. The appearance of the phosphate (P–O) band at approximately 1030 cm^−1^ in the nano-HA-containing scaffolds supported the successful incorporation of hydroxyapatite, consistent with previous reports on calcium-phosphate and PLA/HA materials [20,25]. In contrast, we observed no distinct CHX-specific bands, likely because of its relatively low concentration (0.2%) and the overlap of its characteristic vibrations with the stronger PLA absorption bands [26]. Therefore, the absence of a clearly distinguishable CHX band should not necessarily be interpreted as the absence of the antimicrobial agent within the scaffold. These findings suggest that the developed scaffolds preserved the chemical integrity of PLA while allowing the successful incorporation of nano-HA without inducing substantial adverse chemical interactions.

EDS analysis confirmed the elemental composition of the developed scaffolds and provided additional evidence for the incorporation of nano-HA and CHX into the PLA matrix. As expected, carbon and oxygen were the predominant elements in all groups due to the polymeric nature of PLA. In contrast, phosphorus and calcium were detected in the nano-HA-containing scaffolds, confirming the presence of the bioactive ceramic phase. Similar elemental profiles have been reported for PLA/nano-HA composites and are considered indicative of successful HA incorporation within the polymer matrix [12,27]. Likewise, chlorine was identified in the CHX-containing groups, supporting the presence of the CHX formulation within the scaffolds [26]. Nevertheless, because EDS identifies elemental rather than molecular composition, the detection of chlorine should be considered complementary evidence rather than definitive molecular confirmation of CHX. The elemental composition was consistent with the SEM observations, which showed surface-associated particles in nano-HA-containing membranes, and with the FTIR spectra, which showed the characteristic phosphate absorption band.

The cell adhesion analyses demonstrated that incorporation of nano-HA and, most importantly, CHX did not compromise the overall cytocompatibility of the electrospun PLA scaffolds. CHX-containing membranes did not compromise cell viability after six days of culture. In contrast, the nano-HA-containing membranes showed favorable cell adhesion and metabolic activity during the evaluated culture period. Although CHX has been associated with concentration- and exposure-dependent cytotoxicity, previous studies have demonstrated that its biological effects are strongly influenced by concentration and delivery conditions [28,29]. Similar findings have been reported for PLA/HA composites, where HA can promote favorable cell attachment and interactions due to its osteoconductive properties and the physicochemical characteristics of its calcium phosphate surface [20,27,30]. The maintenance of cell viability in the CHX-containing groups suggests that incorporation of CHX within the PLA scaffold at the selected concentration was compatible with the cellular conditions evaluated in the present study. The cellular response observed in the dual PLA/nano-HA/CHX scaffold suggests that nano-HA and CHX allowed the combination of a bioactive component and an antimicrobial agent without compromising overall cell viability. These findings support the potential application of the developed bilayer membrane for guided bone regeneration.

Several limitations of the present study warrant consideration. Although we performed comprehensive physicochemical and biological characterization of the scaffolds, we did not evaluate their degradation behavior or the release kinetics of the incorporated components. Further studies should therefore more thoroughly investigate scaffold degradation, nano-HA behavior, and CHX release kinetics to characterize the system’s stability and drug-delivery potential. In addition, although the in vitro biocompatibility assays yielded promising results, evaluation in appropriate animal models is necessary to determine the biological response, tissue integration, and degradation behavior under in vivo conditions before considering clinical translation. Furthermore, the antimicrobial performance of the CHX-containing scaffolds should be evaluated to determine whether the incorporated CHX provides effective, sustained local antimicrobial activity. Finally, before suggesting that this prototype can effectively support guided bone regeneration, its pore architecture, degradation behavior, and interfacial stability should be investigated, ideally through in vivo studies.

## 5. Conclusions

This study demonstrated the feasibility of fabricating a bilayer electrospun PLA membrane incorporating nano-HA and CHX within spatially distinct layers. The resulting membrane preserved the characteristic fibrous architecture of electrospun PLA while retaining the morphological and compositional features associated with the nano-HA- and CHX-containing layers. All formulations were successfully physicochemically characterized and maintained in vitro cytocompatibility. Collectively, these findings establish the feasibility of the proposed PLA/nano-HA/CHX architecture as a candidate bilayer platform for further investigation in guided bone regeneration. However, its potential antibacterial activity and osteogenic performance require direct experimental validation.

## Figures and Tables

**Table 1:** Cross-sectional thickness of the electrospun membranes according to formulation. Values are presented as mean ± standard deviation (SD) in µm. Different uppercase letters indicate statistically significant differences between experimental groups according to pairwise Mann–Whitney U tests with Bonferroni correction (*p* < 0.05), following a significant Kruskal–Wallis test. PLA: poly(lactic acid); CHX: chlorhexidine; nano-HA: nano-hydroxyapatite.

Group	Mean (µm) ± SD	Statistical Group
PLA 10%	95.00 ± 5.10	A
PLA 10%—nano—HA 10%	148.30 ± 12.77	B
PLA 10%—CHX 0.2%	82.80 ± 6.78	C
PLA 10%—CHX 0.2%—nano-HA 10%	69.30 ± 2.63	D

**Table 2:** Mean and standard deviation values obtained by DSC. T_g-rel_: enthalpic-relaxation event associated with the PLA glass-transition region. T_cc_: temperature of cold-crystallization. T_m_: melting point. Temperatures are expressed in °C. Within each thermal event (column), different uppercase letters indicate statistically significant differences between groups.

Group	T_g-rel_ Mean ± SD.		T_cc_ Mean ± SD		T_m_ Mean ± SD	
PLA 10%	64.51 ± 0.62	A	104.22 ± 1.20	A	157.04 ± 0.51	A
PLA 10%/CHX 0.2%	65.23 ± 0.23	A	100.10 ± 0.10	B	155.80 ± 0.61	A
PLA 10%/nano-HA 10%	64.51 ± 1.22	A	102.31 ± 1.01	B	155.33 ± 0.09	A
PLA 10%/CHX 0.2%/nano-HA 10%	64.74 ± 0.69	A	99.77 ± 1.02	AB	155.81 ± 0.19	A

**Table 3:** Transition enthalpy (ΔH) for each signal. ΔHg-rel: enthalpy associated with enthalpic relaxation in the glass transition region. ΔHcc: cold-crystallization enthalpy. ΔHm: melting enthalpy. Values are expressed as mean ± standard deviation (SD) in J/g. Within each thermal event (column), different uppercase letters indicate statistically significant differences between groups (*p* < 0.05), whereas groups sharing the same letter are not significantly different.

Group	ΔH_g-rel_ Mean ± SD		ΔH_cc_ Mean ± SD		ΔH_m_ Mean ± SD	
PLA 10%	26.97 ± 5.31	A	88.33 ± 1.58	A	148.33 ± 7.13	A
PLA 10%/CHX 0.2%	28.42 ± 5.57	A	58.62 ± 18.08	AB	165.42 ± 33.52	A
PLA 10%/nano-HA 10%	21.62 ± 2.27	A	25.38 ± 10.07	B	79.23 ± 2.45	B
PLA 10%/CHX 0.2%/nano-HA 10%	28.12 ± 4.8	A	47.49 ± 15.9	B	160.91 ± 12.59	A

**Table 4:** Thermogravimetric analysis of membranes.

Group	Onset Point (T_o_) Mean ± SD	Inflection Point (T_p_) Mean ± SD
PLA 10%	338.70 ± 7.74	366.50 ± 6.23
PLA 10%/CHX 0.2%	327.65 ± 10.92	365.97 ± 4.06
PLA 10%/nano-HA 10%	327.75 ± 12.07	353.68 ± 16.12
PLA 10%/CHX 0.2%/nano-HA 10%	326.89 ± 26.37	349.84 ± 29.15

## Data Availability

All relevant data are within the paper.
